# Cerebellar activation associated with model-based estimation of tool-use consequences

**DOI:** 10.1186/s12993-019-0158-y

**Published:** 2019-04-16

**Authors:** Sayako Ueda, Hiroyuki Sakai, Kenichi Ueno, Kang Cheng, Takatsune Kumada

**Affiliations:** 1 TOYOTA Collaboration Center, RIKEN Center for Brain Science, RIKEN, 2-1 Hirosawa, Wako, Saitama 351-0198 Japan; 2 Toyota Central R&D Laboratories, Nagakute, Japan; 3 Support Unit for Functional Magnetic Resonance Imaging, RIKEN Center for Brain Science, Wako, Japan; 4 Graduate School of Informatics, Kyoto University, Kyoto, Japan

**Keywords:** Cerebellum, Tool use, Internal model, Estimation, fMRI

## Abstract

**Background:**

Dexterous tool use is considered to be underpinned by model-based control relying on acquired internal models of tools. In particular, this is the case in situations where available sensory feedback regarding the consequences of tool use is restricted. In the present study, we conducted an fMRI study to identify cerebellar involvement in model-based estimation of tool-use consequences using tracking tasks with different levels of visual feedback.

**Methods:**

Twenty healthy right-handed adults participated in this study. These participants tracked a moving target on a screen with a cursor controlled by a joystick using their right hand during fMRI scanning. For each trial, the level of visual feedback for cursor position was randomly selected from three task conditions, namely, Precise, Obscure, and No conditions.

**Results:**

A conjunction analysis across all task conditions found extensive activation of the right cerebellum, covering the anterior lobe (lobule V) and inferior posterior lobe (lobule VIII). Also, contrasts among the three task conditions revealed additional significant activation of the left superior posterior lobe (Crus I) in the No compared to the Precise condition. Furthermore, a post hoc psychophysiological interaction analysis revealed conditional modulation of functional coupling between the right, but not the left, cerebellar region and right frontoparietal regions that are involved in self-body perception.

**Conclusions:**

Our data show that the left Crus I is the only region that was more active in a condition where no visual feedback for cursor position was available. This suggests that the left Crus I region plays a role in model-based estimation of tool-use consequences based on an acquired internal model of tools.

**Electronic supplementary material:**

The online version of this article (10.1186/s12993-019-0158-y) contains supplementary material, which is available to authorized users.

## Background

People dexterously use a wide variety of tools. This remarkable ability is not limited to the use of simple hand-held tools such as a hammer or chopsticks, rather, it applies as well to various tools that have a complex input–output relationship. For example, when using a computer mouse, operators must learn a coordinate transformation rule between mouse movement on a desk and cursor position in a computer screen. Also, in the case of operating a car, drivers must learn complicated vehicle dynamics to maneuver the car as intended.

A substantial number of empirical studies suggest that dexterous tool use is underpinned by model-based control relying on acquired internal models of tools. Dingwell et al. [1], for instance, demonstrated that behavioral results of a robot-arm manipulation task are reproduced better by a model-based feedforward controller rather than model-free feedback one. Mah and Mussa-Ivaldi [2] found that inter-task transfer of object manipulation skills is proportional to the similarity of object dynamics between tasks, which suggests that internal models are acquired and reused for object manipulation. Furthermore, using a pole-balancing task in a virtual environment, Mehta and Schaal [3] showed that people can keep balancing a pole even during periods when it was visually occluded. This suggests that sensory feedback regarding the consequences of tool use can be estimated using acquired internal forward models of tools when this feedback is not available.

The cerebellum is a primary neural basis for model-based tool use (see [4], for a review). The pioneering study of Imamizu et al. [5] identified cerebellar activity that is associated with model-based tool use using a computational neuroscience approach. That is, cerebellar activity during a visuomotor tracking task was modeled as the sum of two distinct activities with respectively different time courses during tool-use learning: one of these activity stemmed from a model-based control origin, which was expected to increase with learning; the other originated from tracking errors and was assumed to decrease with learning. Consequently, Imamizu and colleagues were able to successfully isolate cerebellar activity associated with model-based tool use.

Another approach to identification of cerebellar activities associated with model-based tool use may be the imposition of an estimation of tool-use consequences. This is because estimating tool-use consequences relies on an acquired internal forward model of a tool’s input–output relationship. Restricting visual feedback during tool use is a way to impose model-based estimation of tool-use consequences [3]. Indeed, Ogawa and Inui [6] have demonstrated a functional magnetic resonance (fMRI) study using this approach. They explored brain activity in a visuo-motor tracking task, where participants were asked to track a sinusoidally moving target with a visible or invisible mouse cursor. As a result, greater activation in tracking with invisible compared with visible cursor was found in the pre-supplementary motor area, bilateral inferior parietal lobule, and right cuneus, but not in the cerebellum. This implies that the cerebellum has no obvious role in model-based estimation of tool-use consequences. However, there is an alternative explanation for their results. That is, the brain activity they found may be associated with reproduction of predetermined movements because target motion was a regular sinusoidal pattern, hence affording temporal predictability. To appropriately test cerebellar contributions to model-based estimation of tool-use consequences, target movement should be unpredictable.

Therefore, in the present study we aimed to identify cerebellar regions involved in model-based estimation of tool-use consequences which accord with the assumption that restricted visual feedback, regarding tool-use consequences, is compensated on the basis of acquired internal forward models of tools. To achieve this goal, we conducted an fMRI study in which participants tracked a moving target with a cursor controlled by a joystick using the right hand under different levels of visual feedback. More specifically, in one-third of the trials, both target and cursor were easily identifiable and thus there was no need for estimating cursor position. That is, this is the case in the Precise condition, where the demand for model-based estimation of tool-use consequences should be minimized. In another one-third of the trials, the exact location of the cursor was obscured and therefore model-based estimation of cursor position was somewhat helpful for tracking; this refers to the Obscure condition where demand for model-based estimation should be intermediate. In the remaining one-third of trials, no visual feedback for cursor position was provided, namely the No condition; in this condition, the demand for model-based estimation should be maximized. Thereby, we separately identified cerebellar regions associated with common components for joystick control as the conjunction of all conditions and those associated with model-based estimation of tool-use consequences by contrasting conditions according to the order of the level of visual feedback.

## Methods

### Participants

Twenty adults (10 females) with a mean age of 22.9 years (range 20–33 years; SD = 3.4) participated. All were experimentally naïve and had normal or corrected-to-normal vision. They received pay for their participation. All participants were strictly right-handed as assessed using the Edinburgh Handedness Inventory [7]. In this inventory, the perfect right-handed score is + 100 and all participants scored + 100. Written informed consent was obtained in accordance with a protocol approved by the RIKEN Research Ethics Committee.

### Apparatus

MRI acquisition was conducted on a 4-T whole-body MRI system (Agilent Technologies, Santa Clara, CA, USA) equipped with a head gradient system (Magnex Scientific Ltd., Abingdon, UK). A transverse electromagnetic volume transmitter coil (Takashima Seisakusho, Tokyo, Japan) and a 16-array receiver coil (Nova Medical Inc., Wilmington, MA, USA) were employed to acquire anatomical and functional brain images.

Stimuli were viewed through an optic-fiber goggle system (resolution, 800 × 600; field of view, 24° × 18°; refresh rate, 60 Hz) and were controlled by a computer using the Psychophysics Toolbox [8, 9]. Eye reflection was corrected for each participant by adjusting refractive correction lenses built into the goggles. The left eye of participants was monitored during experiments to check general states.

An MRI-compatible joystick (RTC Joystick, Resonance Technology Inc.) was used as an input device. The joystick was placed on a platform that was affixed to the scanner bed. The platform position was adjusted on an individual basis. Participants operated the joystick using the right hand. For tracking tasks, joystick tilt angle was converted to a cursor position on the goggle screen. The neutral position of the joystick corresponded to the origin of the cursor position (the screen center); joystick angle was proportional to cursor position. This cursor position control process was done at an interval of 1/60 s, using the Psychophysics Toolbox. Note that participants were not able to see their hand in the scanner; therefore, vision of hand and joystick was not available during tasks. Participants had to control the cursor without any visual feedback.

### Task

**Fig. 1:**
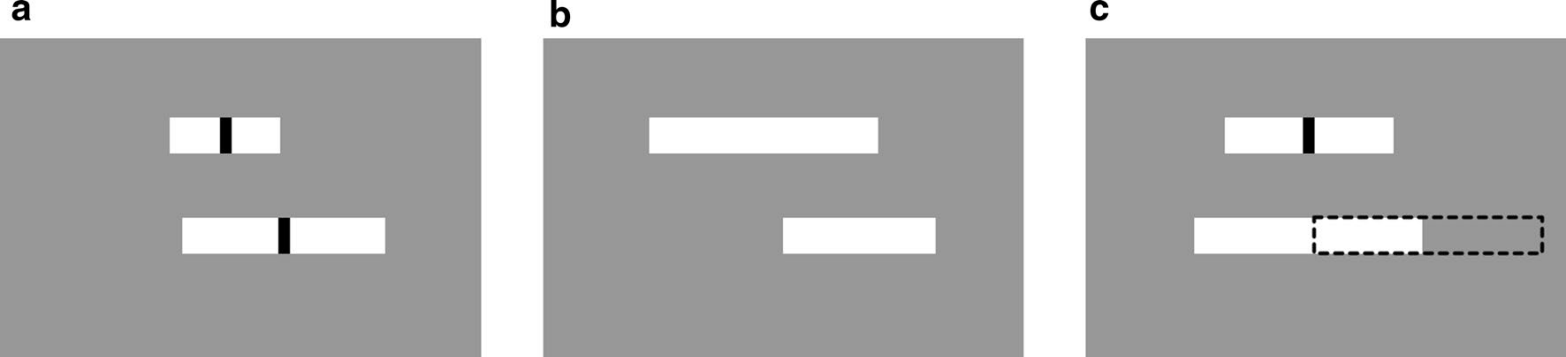
Task conditions. The target and cursor are the upper and lower white bars, respectively. Their lengths were independently varied over time. The target horizontally moved unpredictably. Participants were instructed to track the center of target with the center of cursor. In the Precise condition (**a**), visual markers (black lines) were presented at the centers of both target and cursor and therefore there was no need to estimate cursor position. In the Obscure condition (**b**), no visual markers were provided and therefore estimating cursor position was somewhat helpful for tracking. In the No condition (**c**), while the visual marker was provided only for the target, the actual cursor (dotted bar) was invisible; instead, a fake cursor (white bar) was displayed which automatically and completely followed the target independently of input signals from the joystick. Therefore, positions of the actual and fake cursors were independent of each other and the demand for estimating position of the actual cursor was maximized

Figure 1 illustrates task conditions with different levels of visual feedback for cursor position (Precise, Obscure, and No). In all conditions, both target and cursor were white bars presented on a gray background. The target was located on the upper third of the screen, while the cursor was located 30 pixels below the target. The target moved smoothly along the horizontal axis according to the sum of six sinusoidal functions with different frequencies; and their lengths were independently varied over time as the sum of two sinusoidal functions with different frequencies (range 61–235 pixels). In the Precise condition, in order to provide precise feedback information, thin vertical black lines (5 × 25 pixels) were always overlaid at the center of both target and cursor as visual markers (Fig. 1a). In this condition, participants could use the gap distance between black lines of the two white bars for cursor control. By contrast, in the Obscure condition, no center marker was provided for either the target or the cursor (Fig. 1b). And, in the No condition, while the black line was presented at the center of the target, the cursor automatically, and completely, followed the target independently of input signals from the joystick (Fig. 1c).

### Procedure

**Fig. 2:**
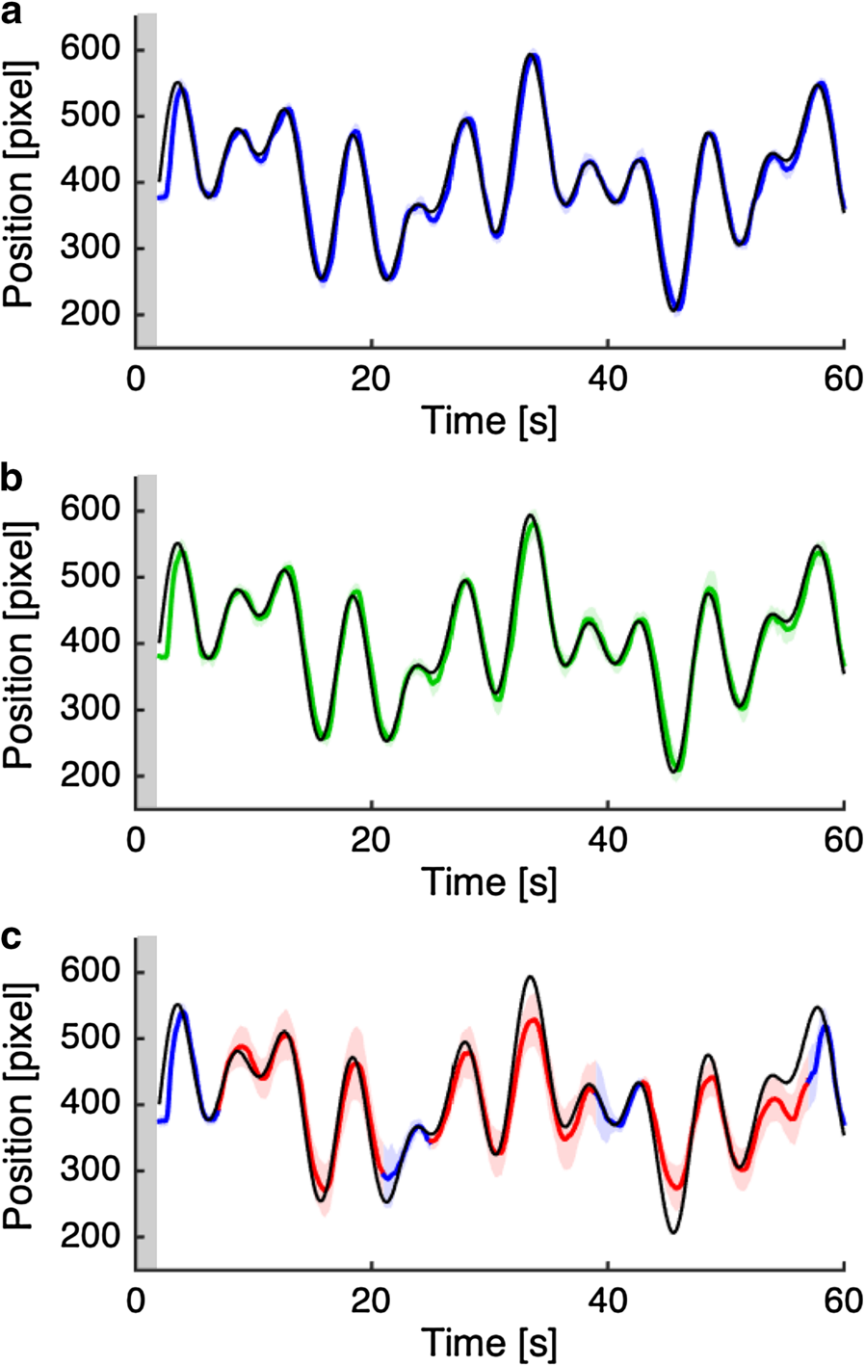
Examples of target and cursor movements in the Precise (**a**, blue line), Obscure (**b**, green line), and No (**c**, red line) conditions. Thin black lines are target trajectories and colored lines are the mean cursor movements across participants. Colored shaded areas represent the standard deviation of the mean. Gray shaded areas represent periods of visual instruction for specifying task conditions. Note that in the No condition (**c**), visual feedback for cursor position was occasionally provided as indicated by blue lines. These periods in the No condition was treated as the Precise condition in data analysis

Participants received sufficient practice on our tracking tasks inside the magnet for enabling comprehension of task instructions (five blocks for each task condition; practice session). More importantly, they could learn the input–output relationship between joystick and cursor movements. Participants were then required to perform three fMRI runs (fMRI scanning session). Each run comprised six task blocks of 60-s duration, which alternated with 30-s duration fixation periods. The three task conditions were pseudo-randomly interleaved within each run. At the beginning of each task block, visual instruction (2-s duration) was provided to specify the task condition. The Precise and Obscure conditions continued for the rest of the blocks (58-s duration). The No condition of 14-s duration was alternated with brief periods (3, 4 or 5 s) of the Precise condition (Fig. 2c). This was designed to prevent participants from giving up on task performance. Note that a constant target trajectory was repeatedly presented throughout the practice session; in contrast, in the fMRI scanning session, another six target trajectories were used once for each run (comprising six task blocks) and each trajectory was presented once for each task condition across the three runs.

### MRI acquisition

Prior to fMRI data collection, a high-resolution T1-weighted anatomical image was acquired using a 3D MPRAGE sequence for each participant. The imaging parameters were as follows: inversion time = 0.5 s, repetition time (TR) = 11 ms, echo time (TE) = 4 ms, flip angle (FA) = 11 deg, field of view (FOV) = 256 × 256 × 180 mm, matrix size = 256 × 256 × 180. Then, fMRI data during tracking tasks were scanned using a gradient echo T2*-weighted two-segmented echo-planar imaging (EPI) sequence with a volume TR of 3000 ms, a TE of 35 ms, an FA of 84°, an FOV of 192 × 192 mm, a matrix size of 64 × 64, and 36 contiguous 4-mm thickness axial slices. For each of the three runs, a total of 95 EPI volumes were collected. During fMRI scanning, respiratory and cardiac signals were simultaneously recorded using a pressure sensor and a pulse oximeter, respectively. These signals were used to remove physiological fluctuations from the EPI images [10].

### Analysis of behavioral data

Tracking performance was measured using tracking error defined as the root mean square of position error (the distance between the centers of target and cursor). In this regard, the periods when visual feedback regarding cursor position was provided in the No condition were included in the calculation of tracking error for the Precise condition. Therefore, tracking error in the No condition were computed only from the invisible periods of the cursor. In addition, to specify a chance level of tracking performance, we calculated tracking error by shuffling the combinations of cursor and target trajectories across blocks. The result was labeled the Shuffle condition for the sake of convenience. Larger tracking error implies less accurate tracking.

### Analysis of fMRI data

Image preprocessing was performed using the SPM12 software package (http://www.fil.ion.ucl.ac.uk/spm/). For each participant, EPI images were spatially realigned for motion correction and coregistered to the anatomical image. Thereafter, the anatomical image was segmented into gray matter (GM), white matter (WM) and cerebro-spinal fluid (CSF) and normalized to Montreal Neurological Institute (MNI) space to obtain a deformation field, using the CAT12 toolbox. By applying the deformation field, the coregistered EPI images were warped to MNI space and resliced to an isotropic voxel size of 2 × 2 × 2 mm^3^. The warped EPI images were smoothed with a 6-mm full-width at half-maximum isotropic Gaussian kernel.

For first-level analysis, a time series of EPI images from each participant were regressed voxelwise using a boxcar function convolved with the default hemodynamic response function in SPM12. Each task condition (i.e., Precise, Obscure, or No condition) was modeled as a separate regressor. Note that inserted periods in which visual feedback regarding cursor position was provided during the No condition were incorporated into a regressor for the Precise condition. In addition, visual instructions at the onsets of task blocks were also modeled as a nuisance regressor. A temporal high-pass filter with a cut-off frequency of 1/570 Hz was used for baseline correction, hence task-related signals were not filtered out. Consequently, contrast images were calculated for effects of interest (i.e., individual task conditions).

Subsequently, the first-level contrast images from all participants were introduced in the second-level random-effect analysis to allow for population inference. To determine task-related cerebellar activation, all the contrast images were entered into a full factorial general linear model with task condition as a factor and statistically examined voxel-wise within a cerebellar mask that was constructed using the MNI structural atlas in FSL (http://www.fmrib.ox.ac.uk/fsl). In order to identify cerebellar activation associated with model-based estimation of tool-use consequences, each task condition was contrasted with each of the other two task conditions. In addition, conjunction analysis was conducted to delineate cerebellar regions commonly activated across the three task conditions. Statistical criteria were set at *p* < 0.05 family-wise error (FWE) corrected for multiple comparisons at the cluster level with a voxel level threshold of *p* < 0.001 uncorrected. Brain regions showing significant activation were localized and visualized using a flat representation specialized for the human cerebellum [11]. Note that cerebral activation was also examined with similar procedures; their results appear in Additional file 1.

Moreover, a post hoc PPI analysis was performed to assess cerebellar functional connectivity associated with model-based estimation of tool-use consequences. This entailed reliance upon the default pipeline for a generalized form of context-dependent PPI analysis [12] integrated in the CONN toolbox (http://www.nitrc.org/projects/conn). Seed regions were cerebellar regions identified in the above mentioned activation analysis (i.e., contrasts between conditions and conjunction across the three conditions). Functional connectivity of each seed region with the rest of the (whole) brain was compared voxel-wise among the three task conditions. Statistical criteria were set at FWE corrected *p* < 0.05 at the cluster level with a voxel level threshold of *p* < 0.001 uncorrected.

## Results

One male participant was excluded from analyses because he failed to stay awake during fMRI scanning. Therefore, the following analyses were performed on data from the remaining 19 participants.

### Tracking performance

Figure 2 illustrates examples of target and cursor movements in the fMRI scanning session. On average, participants were able to accurately track a moving target in the Precise (Fig. 2a) and Obscure (Fig. 2b) conditions. More importantly, even in the No condition (Fig. 2c), it appeared that cursor movement followed a target trajectory, although participants tended to somewhat overestimate cursor movement.

**Fig. 3:**
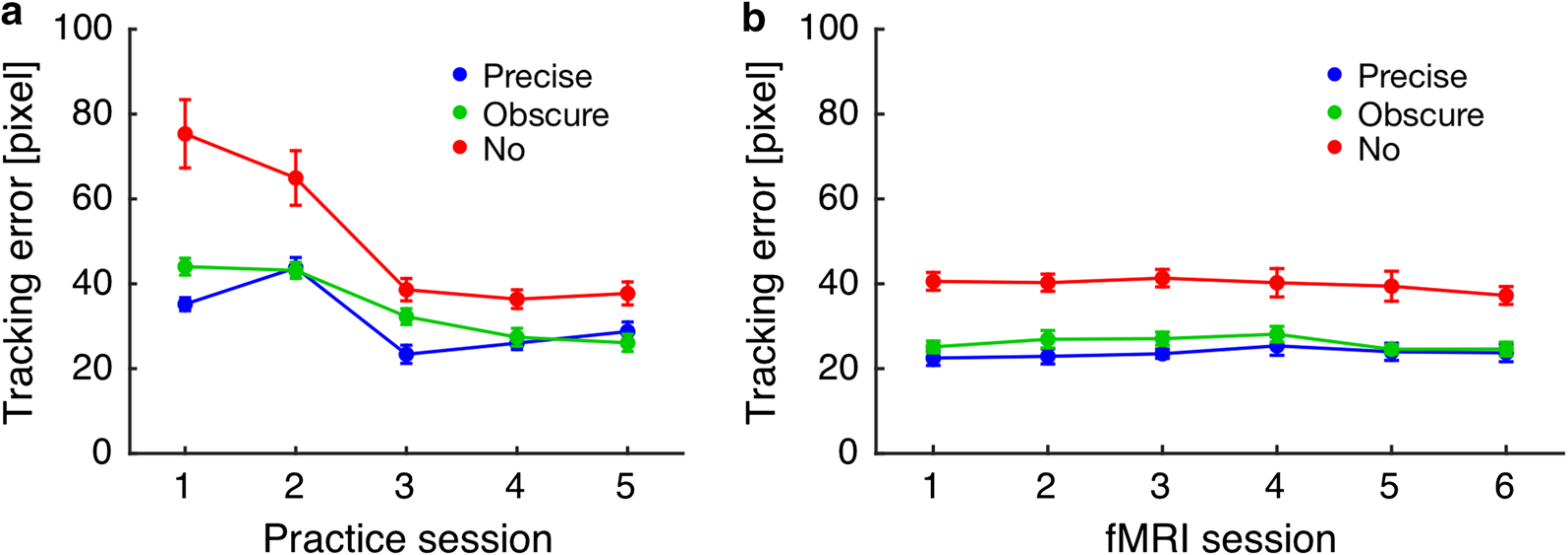
Tracking error as a function of trial for each task condition. Tracking error tended to decrease and reach a plateau during the practice session (**a**), and seemed to be stable during the fMRI scanning session (**b**). Error bars represent 95% confidence intervals

Figure 3 shows changes in tracking errors during the practice and fMRI scanning sessions. Tracking errors tended to decrease and reach a plateau during the practice session (Fig. 3a), and they seemed stable during the fMRI scanning session (Fig. 3b). To assess these trends statistically, we performed a repeated measures analysis of variance (ANOVA) with factors of task condition and block, separately for the practice and fMRI scanning sessions. We found an expected main effect of block in the practice session (*F*(4,72) = 47.48, *p* < 0.001, $\eta_{p}^{ 2}$ = 0.73), but not in the fMRI scanning session (*F*(5,90) = 2.03, *p *= 0.08, $\eta_{p}^{ 2}$ = 0.10). The main effect of task condition was significant both in the practice session (*F*(2,36) = 127.91, *p* < 0.001, $\eta_{p}^{ 2}$ = 0.88) and in the fMRI scanning session (*F*(2, 36) = 202.19, *p* < 0.001, $\eta_{p}^{ 2}$ = 0.99). The interaction of task condition and block was significant in the practice session (*F*(8, 144) = 15.30, *p* < 0.001, $\eta_{p}^{ 2}$ = 0.46), but it was not significant in the fMRI scanning session (*F*(10, 180) = 1.51, *p* = 0.14, $\eta_{p}^{ 2}$ = 0.08).

**Fig. 4:**
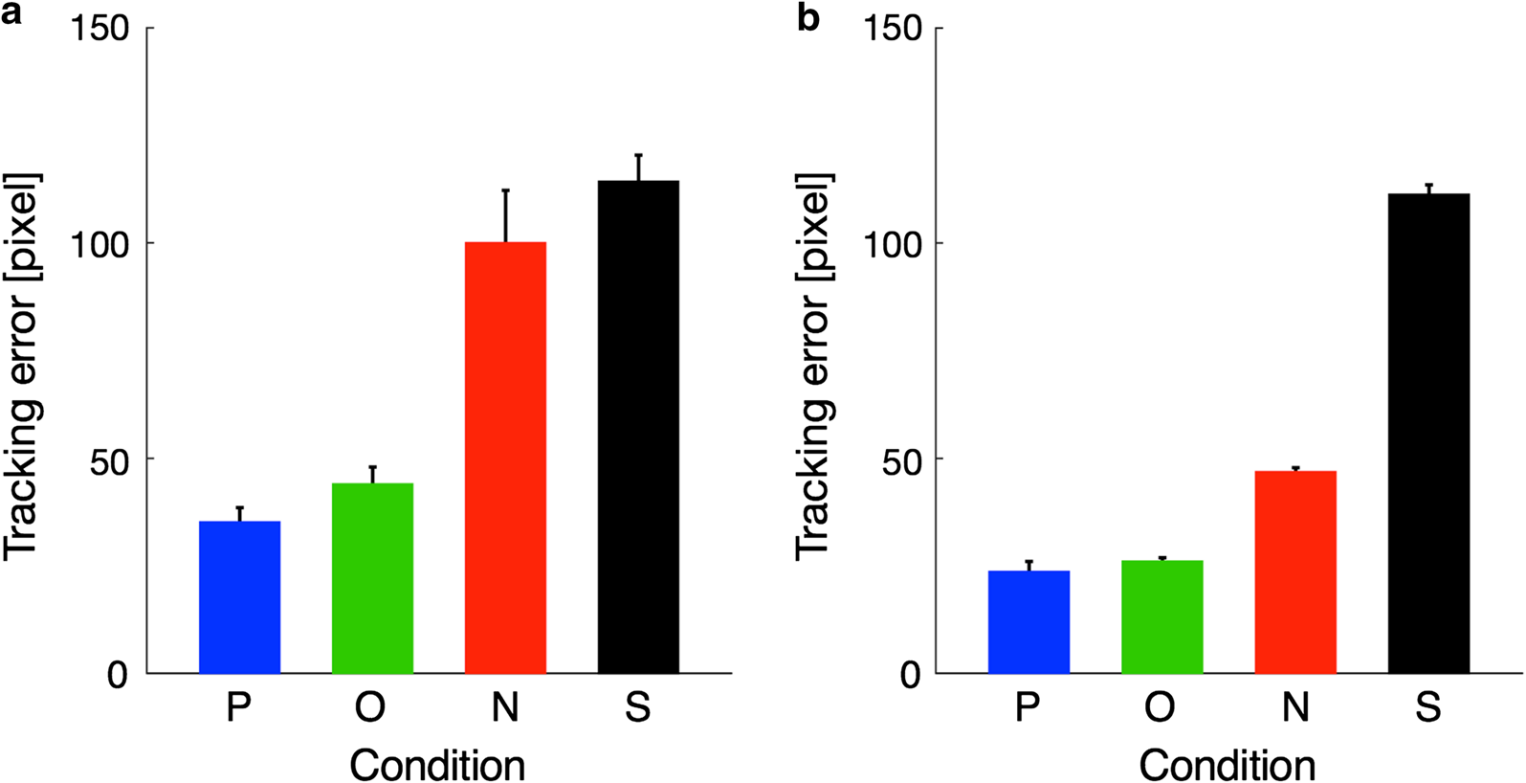
The tracking error in the first block of the practice session (**a**) and the mean tracking error in the fMRI scanning session (**b**). In the fMRI scanning session, for each participant, tracking error was averaged across task blocks for each task condition. P, O, and N denote the Precise, Obscure, and No conditions, respectively. In addition, the chance level of tracking error was computed by shuffling the combinations of the cursor trajectories and the target trajectories across blocks, which was labeled as the shuffle (S) condition for the sake of convenience. Error bars represent 95% confidence intervals

Figure 4a shows the tracking errors for each task condition in the first block of the practice session and Fig. 4b shows the mean tracking error for each task condition in the fMRI scanning session. A one-way repeated measures ANOVA was performed separately for the first block of the practice session and for the fMRI scanning session. These analyses resulted in significant main effect of task condition in the first block of the practice session (*F*(3,54) = 65.06, *p* < 0.001, $\eta_{p}^{ 2}$ = 0.78) and in the fMRI scanning session (*F*(3,54) = 1211.79, *p* < 0.001, $\eta_{p}^{ 2}$ = 0.99). A post hoc multiple comparisons test using Shaffer’s modified sequentially rejective Bonferroni procedure revealed an increase in tracking error with the assumed order of visual feedback for cursor position, i.e., Precise < Obscure in the first block of the practice session (*t*(18) = 7.65, *p* < 0.001, *d* = 1.75) and in the fMRI scanning session (*t*(18) = 6.26, *p* < 0.001, *d* = 1.44), and Obscure < No in the first block of the practice session (*t*(18) = 6.05, *p* < 0.001, *d* = 1.38) and in the fMRI scanning session (*t*(18) = 14.08, *p* < 0.001, *d* = 3.23). Most importantly, tracking errors in the No condition were not different from those observed in the Shuffle condition in the first block of the practice session (*t*(18) = 1.37, *p* = 0.19, *d* = 0.31), but these errors in the No condition were significantly fewer than in the Shuffle condition for the fMRI scanning session (*t*(18) = 25.74, *p* < 0.001, *d* = 5.90). This means that participants began to control the (invisible) cursor more accurately, thus leading to above chance level performance within the practice session; this suggests that participants acquired an internal model concerning transformation from joystick angle to cursor position through the practice session and used it to estimate cursor position in the No condition.

### Cerebellar activation

**Fig. 5:**
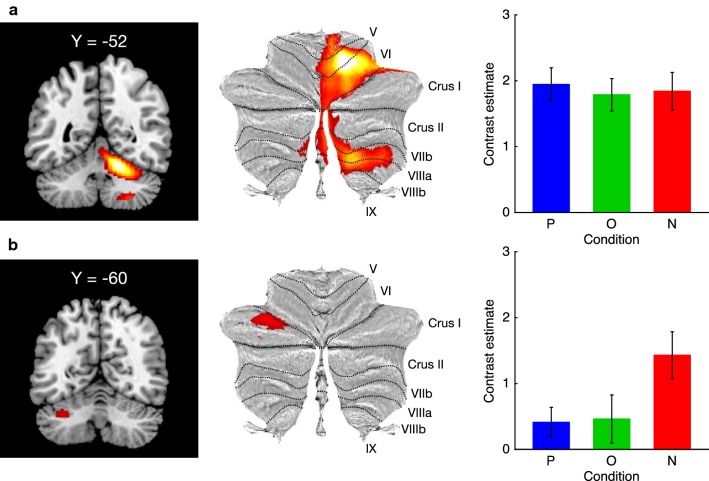
Cerebellar regions showing greater activation during tracking tasks. The right cerebellar region (**a**) were found to be commonly activated in the three task conditions. In contrast, another region in the left cerebellum (**b**) were more active in No compared to Precise condition. Statistical criteria was set at *p* < 0.05 family-wise error (FWE) corrected for multiple comparisons at the cluster level with a voxel level threshold of *p* < 0.001 uncorrected

Table 1 summarizes significant cerebellar activation. The conjunction analysis across the three task conditions reveals extensive activation of the right cerebellum (Fig. 5a). Peak loci were found in the anterior (lobule V) and inferior posterior (lobule VIII) lobes. In this cluster, no significant differences in mean beta estimates were evident among the task conditions (a one-way repeated measures ANOVA, *F*(2,36) = 0.45, *p* = 0.64, $\eta_{p}^{ 2}$ = 0.02; Fig. 5a). Another region in the left cerebellum was found to be more active in producing a comparison of No > Precise. The peak locus was in the superior posterior lobe (Crus I) (Fig. 5b). No other regions were identified in any contrasts of interest. An ANOVA on mean beta estimates within this cluster revealed a significant main effect of task condition (*F*(2,36) = 8.62, *p* < 0.001, $\eta_{p}^{ 2}$ = 0.32); in addition, two post hoc multiple, comparison tests revealed greater activation in the No condition compared to the Precise (*t*(18) = 4.13, *p* < 0.001, *d* = 0.95) and Obscure (*t*(18) = 3.23, *p* < 0.005, *d* = 0.74) conditions (Shaffer’s modified sequentially rejection Bonferroni procedure; Fig. 5b). These results suggest that a distinct region in the left cerebellum is associated with model-based estimation of cursor position.

**Table 1:** Loci of significant cerebellar activation

Region	Peak coordinates			T-score	Extent
	x	y	z		
*Conjunction*					
R lobule V	18	− 52	− 22	11.42	2655
R lobule VIII	16	− 64	− 48	7.17	
*No *>* Precise*					
L Crus I	− 34	− 60	− 32	4.15	117

Peak coordinates are given in the Montreal Neurological Institute space. Statistical criteria was set at *p* < 0.05 family-wise error (FWE) corrected for multiple comparisons at the cluster level with a voxel level threshold of *p* < 0.001 uncorrected. L and R denote left and right hemispheres, respectively

**Fig. 6:**
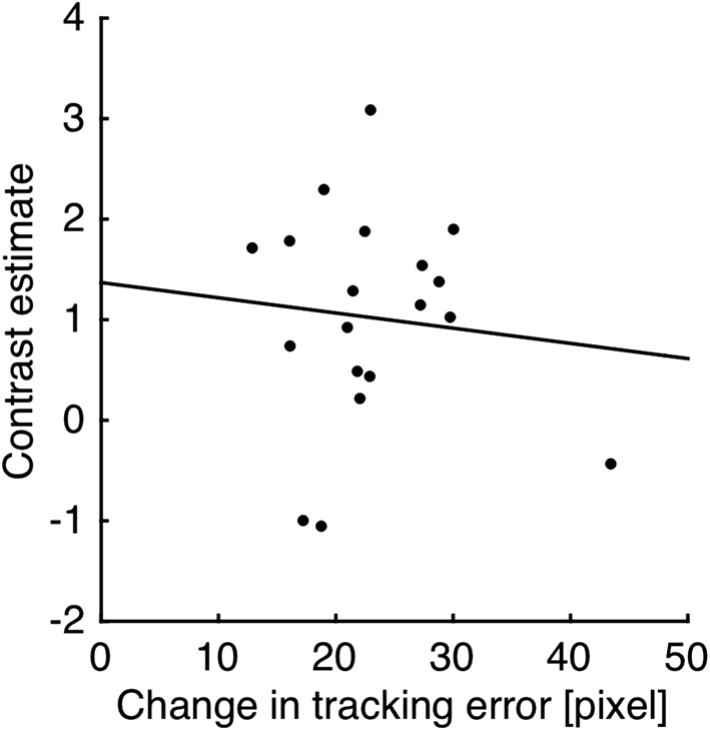
Correlation of cerebellar activation with tracking error. Region of interest was the cluster in which significant activation was found in the No compared to the Precise condition (Fig. 5b). Note that both contrast estimate and tracking error were differences between those in the No condition and those in the Precise condition

Nevertheless, behavioral performance data showed that tracking errors were most prevalent in the No condition relative to the other two conditions. Therefore, one may question whether task difficulty might be a confounding factor. If so, then the left cerebellar activations observed here could be explained as increased tracking error. Figure 6 shows scatter diagrams of increased tracking error (No vs Precise) and contrast estimate for the significant cluster in the left cerebellum. There was no significant correlation between increased tracking error and contrast estimate of the left cerebellum (*r* = − 0.10, *p* = 0.69). This suggests that the left cerebellar activation observed was not simply attributable to increased tracking error and task difficulty in the No condition.

### Cerebello-cortical functional coupling

**Fig. 7:**
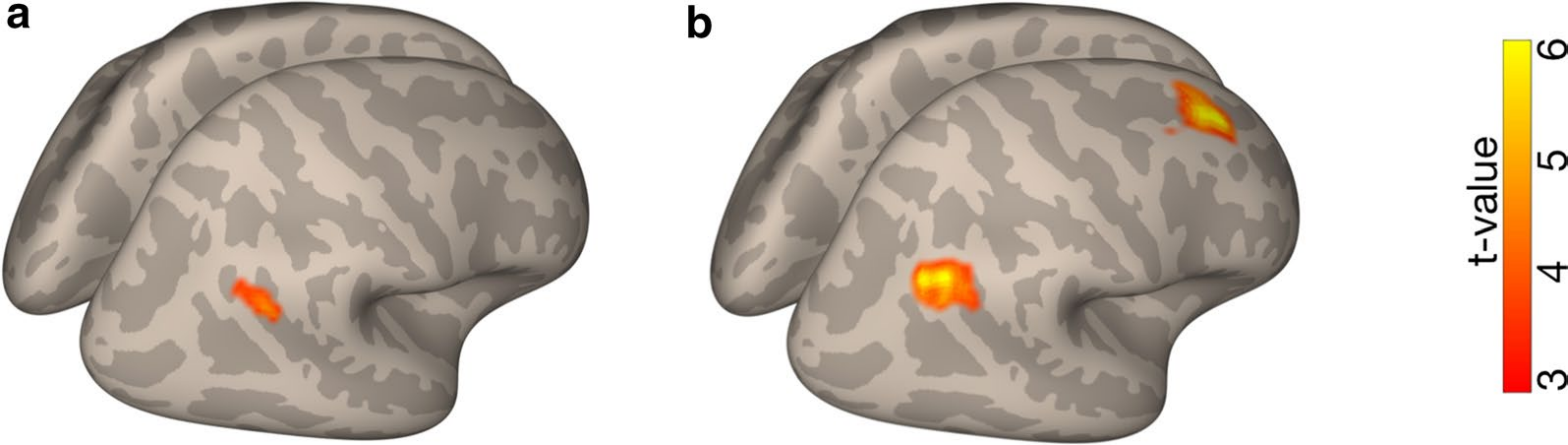
Regions showing conditional modulation of cerebello-cortical functional coupling. The seed region was the right cerebellar cluster in which significant activation was found in conjunction analysis (Fig. 5a). Functional coupling of the right cerebellar region was greater with the angular gyrus in the Obscure compared to the Precise condition (**a**). In addition, the right cerebellar region additionally showed greater functional coupling with the right prefrontal cortex, as well as with the right angular gyrus in the No compared to the Precise condition (**b**). Statistical criteria was set at FWE corrected *p* < 0.05 at the cluster level with a voxel level threshold of *p* < 0.001 uncorrected

A post hoc PPI analysis found conditional modulation of cerebello-cortical functional coupling (Table 2). Specifically, the right cerebellar region (Fig. 5a) showed significantly stronger functional connectivity with the right angular gyrus (AG) in the Obscure when compared with the Precise condition (Fig. 7a). In the contrast of No > Precise conditions, the right cerebellar region also showed stronger functional connectivity with the right prefrontal cortex (PFC), as well as with the right AG (Fig. 7b). In contrast, no significant conditional modulation of functional coupling was found when the left cerebellar region (Fig. 5b) was used as a seed. These results suggest that functional coupling between the right cerebellar region and right frontoparietal regions was modulated depending on demand for model-based estimation of cursor position.

**Table 2:** Loci for significant functional coupling of the right cerebellar region commonly activated in all task conditions

Region	Peak coordinates			T-score	Extent
	x	y	z		
*Obscure *>* Precise*					
R angular gyrus	40	− 54	30	6.49	195
*No *>* Precise*					
R angular gyrus	44	− 70	40	6.02	497
R prefrontal cortex	26	22	34	7.92	458

Peak coordinates are given in the Montreal Neurological Institute space. Statistical criteria was set at FWE corrected *p* < 0.05 at the cluster level with a voxel level threshold of *p* < 0.001 uncorrected. L and R denote left and right hemispheres, respectively

## Discussion

In the current study, we aimed to identify cerebellar activation associated with model-based estimation of tool-use consequences. To this end, we conducted fMRI during tracking tasks with different levels of visual feedback for cursor position. Consequently, we found that an extensive region in the right cerebellum (lobules V and VIII) was commonly activated in all task conditions, and that a distinct region in the left cerebellum (Crus I) was additionally engaged only when no visual feedback for cursor position was available. Furthermore, we found conditional modulation of functional coupling between the right, but not the left, cerebellar region and right frontoparietal regions.

Our data suggest that the left Crus I region is associated with model-based estimation of tool-use consequences. As the behavioral data have demonstrated, participants could reasonably control the cursor even when no visual feedback for cursor position was available (i.e., the No condition). In addition, unlike a previous study showing no cerebellar involvement in a similar experimental set-up [6], in the present study, target movements were unpredictable. This suggests that tracking in the No condition may be governed by estimating cursor position based on an acquired internal forward model of input–output relationship of the joystick. Additionally, the left Crus I was the only region that showed increased activity in the No condition. Thus, it is plausible to conclude that the left Crus I region plays a role in model-based estimation of tool-use consequences.

This conclusion is in line with previous findings regarding a functional role of the left Crus I. For example, neuroimaging studies found that the left Crus I is activated during mental rotation tasks, which requires participants to estimate a rotated visual object based on a three-dimensional rigid model of the object [13–15]. Picazio et al. [16] have shown that disturbing the activity of the left cerebellar hemisphere by continuous theta burst stimulation induces slower response times in a mental rotation task. These findings suggest that the left Crus I is involved in model-based estimation of the consequences of object manipulation. Moreover, a recent exploratory voxel-based morphometry study reported that car drivers have greater gray matter volume in the left cerebellar hemisphere (Crus I/II) than non-drivers [17]. A plausible interpretation of this volumetric change associated with car driving experiences draws upon the assumption that drivers have learned and acquired to operate internal models of complex car dynamics. This is also consistent with our conclusion on a functional role of the left Crus I in model-based estimation of tool-use consequences.

The right cerebellar region (lobules V and VIII) commonly activated in all the three task conditions may be involved in another functional component of tool use. The most reasonable candidate for such a functional component is the right (ipsilateral) hand control for joystick operation, which was shared in the three task conditions. In fact, the right cerebellar region observed largely overlapped the descriptions of the cerebellar sensorimotor homunculi [18], which have been reported in a substantial number of studies as linked to ipsilateral hand movements [13, 14, 19, 20]. Thus, we conclude that activation in the right cerebellar region is associated with the ipsilateral hand control during tool use. Note, however, that this does not mean that the right cerebellar region during tool use is associated with hand movement per se. Rather, it is plausible to assume that motor control for hand movement is composed of several complex model-based processes including motor command generation based on an internal inverse model of the hand and a predictive mode involving sensory consequences based on an internal forward model of the hand (see [21], for a review).

Our exploratory PPI analysis supports this notion of a functional role for the right cerebellar region during tool use. This is because the right cerebellar region showed increased functional connectivity with right parietal and frontal regions (AG and PFC), depending on demand for model-based estimation of cursor position. The right AG is known to play a role in self-body perception. Blanke et al. [22], for example, have demonstrated that focal electrical stimulation to the right AG induces out-of-body experiences. Farrer et al. [23] argued, based on differences in activation between normal subjects and patients with schizophrenia, that the right AG is crucial for recognition of own actions. The right PFC is also considered to play a critical role in self-body perception. For example, Platek et al. [24] found that increased activation of the right PFC was associated with self-face recognition. In addition, the right PFC damage is commonly observed in patients suffering from impaired self-face recognition [25, 26]. These facts may provide an interpretation that tight coupling of the right cerebellar region with the right frontoparietal regions reflect increased demand for self-body perception. This suggests that when visual feedback for cursor position is less available, the use of proprioceptive information regarding right hand posture becomes essential to control joystick angle for tracking, as well as for model-based estimation of tool-use consequences. However, at present, this remains only a possible interpretation based on reverse inference. To specify the functional roles of tight coupling between the right cerebellum and the right frontoparietal regions in tool use remains an open question for future research.

In conclusion, the present findings suggest that the left Crus I is uniquely associated with model-based estimation of tool-use consequences. This region may be recruited by the demands for estimating tool-use consequences even when the tool involved is operated by the contralateral hand. However, this conclusion has limitations in that we cannot rule out the possibility of involvement of left Crus I in visual imagery of tool-use consequences. In the present experimental set-up, although model-based estimation of cursor position is a prerequisite for its visual imagery, activation in the left Crus I could be associated only with visual imagery rather than model-based estimation. Further studies with more sophisticated experimental designs and analytical methods are needed to clearly dissociate model-based estimation process for tool-use consequences from its visual imagery.

## Additional file

**Additional file 1: Figure S1.** Cerebral activation.

## Abbreviations

AG: angular gyrus
PFC: prefrontal cortex
