# Supplementary material for: Cerebellar activation associated with model-based estimation of tool-use consequences
Source: Behav Brain Funct. 2019 Apr 16;15:8. doi: 10.1186/s12993-019-0158-y (PMC6469048; doi:10.1186/s12993-019-0158-y)
Supplement: Supplementary file 1 — Additional file 1: Figure S1. Cerebral activation. [file 12993_2019_158_MOESM1_ESM.docx]

**Additional file 1**


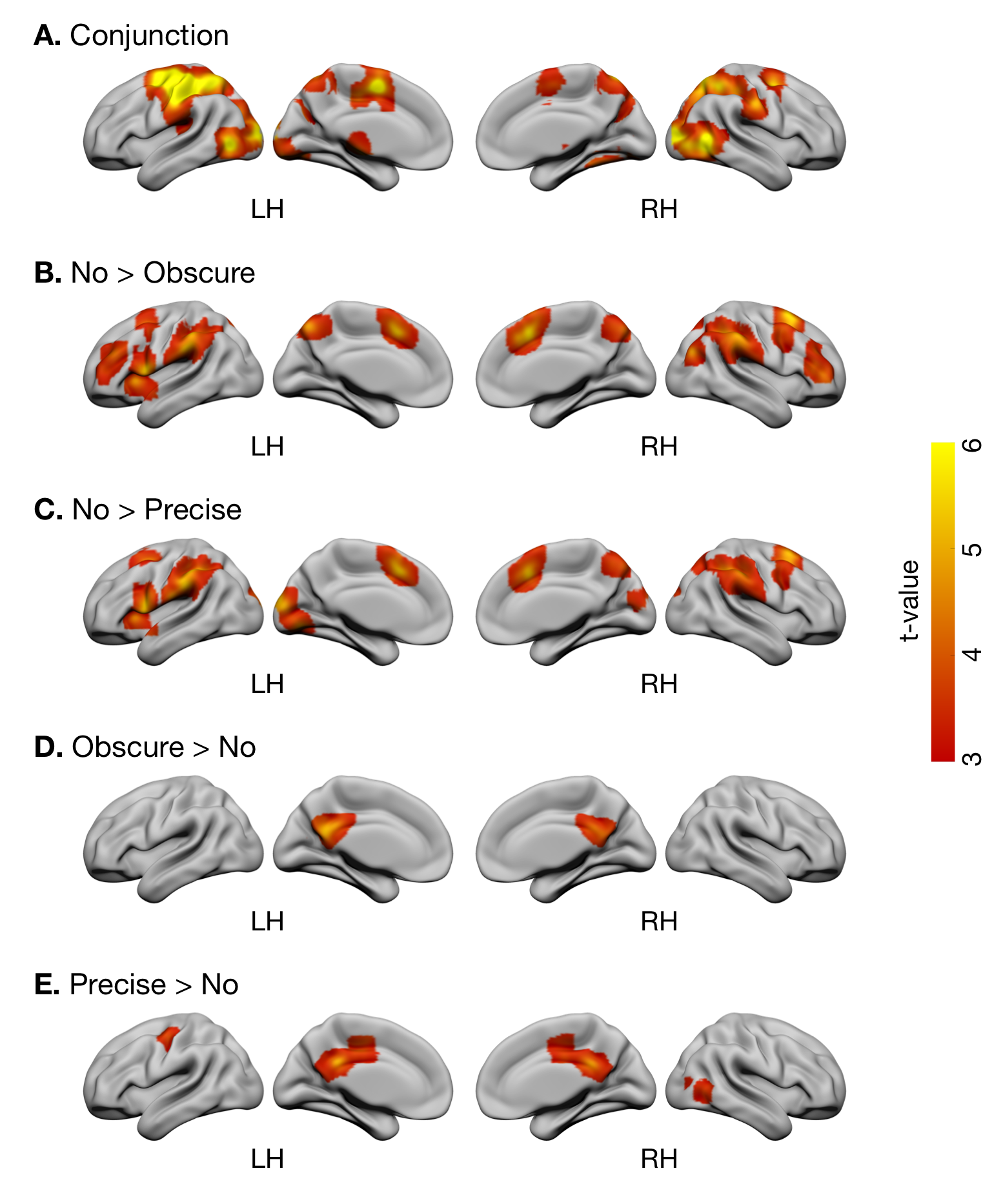


**Fig. S1**  Cerebral activation. A conjunction analysis of all task conditions revealed significant activation in the bilateral occipital, parietal, and frontal cortices, which was at least partially overlapped with regions involved in predicting sensory consequences of body movements [27]. This makes sense because internal model-based control of hand movements is crucial for controlling the joystick in the present tracking tasks, regardless of the level of visual feedback for cursor position. Meanwhile, contrasts between task conditions revealed that reciprocal activation and deactivation of extrinsic mode network (EMN) and default mode network (DMN), depending on the assumed demand for estimating tool-use consequences (i.e., cursor position). In the No condition compared to the other two conditions, significant activation was found in parietal regions (including the precuneus, angular gyrus, and supramarginal gyrus), medial prefrontal regions (including the anterior cingulate cortex and supplementary motor area), and lateral prefrontal regions (including the middle and inferior frontal gyri). These areas indeed constitute a non-specific task-dependent EMN [28]. In contrast, significant deactivation was found in the posterior cingulate cortex, which is a hub of the DMN [29]. Since both EMN activation [28] and DMN deactivation [30] can be induced by increased cognitive load, these cerebral activation patterns are consistent with increased task difficulty in the No condition (Fig. 4B). Statistical criteria was set at FWE-corrected *p* < 0.05 for multiple comparisons at the cluster level with a voxel level threshold of *p* < 0.001 uncorrected.

**Reference**

1. Blakemore SJ, Sirigu A. Action prediction in the cerebellum and in the parietal lobe. Exp Brain Res. 2003;153(2):239–45. https://doi.org/10.1007/s00221-003-1597-z.
2. Hugdahl K, Raichle ME, Mitra A, Specht K. On the existence of a generalized non-specific task-dependent network. Front Hum Neurosci. 2015;9:430. https://doi.org/10.3389/fnhum.2015.00430.
3. Fransson P, Marrelec G. The precuneus/posterior cingulate cortex plays a pivotal role in the default mode network: evidence from a partial correlation network analysis. Neuroimage. 2008;42(3):1178–84. https://doi.org/10.1016/j.neuroimage.2008.05.059.
4. Uchiyama Y, Toyoda H, Sakai H, Shin D, Ebe K, Sadato N. Suppression of brain activity related to a car-following task with an auditory task: an fMRI study. Transp Res F. 2012;15(1):25–37. https://doi.org/10.1016/j.trf.2011.11.002.
